# Continuous Glucose Monitoring as a Matter of Justice

**DOI:** 10.1007/s10730-020-09413-9

**Published:** 2020-05-21

**Authors:** Steven R. Kraaijeveld

**Affiliations:** Wageningen University & Research, Wageningen, The Netherlands

**Keywords:** Continuous glucose monitoring, Justice, Type 1 diabetes, Diabetes mellitus, Health inequality, Access to health care

## Abstract

Type 1 diabetes (T1D) is a chronic illness that requires intensive lifelong management of blood glucose concentrations by means of external insulin administration. There have been substantial developments in the ways of measuring glucose levels, which is crucial to T1D self-management. Recently, continuous glucose monitoring (CGM) has allowed people with T1D to keep track of their blood glucose levels in near real-time. These devices have alarms that warn users about potentially dangerous blood glucose trends, which can often be shared with ther people. CGM is consistently associated with improved glycemic control and reduced hypoglycemia and is currently recommended by doctors. However, due to the costs of CGM, only those who qualify for hospital provision or those who can personally afford it are able to use it, which excludes many people. In this paper, I argue that unequal access to CGM results in: (1) unjust health inequalities, (2) relational injustice, (3) injustice with regard to agency and autonomy, and (4) epistemic injustice. These considerations provide prima facie moral reasons why all people with T1D should have access to CGM technology. I discuss the specific case of CGM policy in the Netherlands, which currently only provides coverage for a small group of people with T1D, and argue that, especially with additional considerations of cost-effectiveness, the Dutch government ought to include CGM in basic health care insurance for all people with T1D.

## Background

Type 1 diabetes (T1D) is a chronic autoimmune condition in which the pancreas produces insufficient insulin or none at all. Given that insulin is necessary for glucose to enter cells and thus to allow the body to function, people with T1D must regulate insulin intake themselves by means of extraneous sources of insulin.^1^ There is currently no cure for T1D—it requires lifelong management, which can be a highly demanding task. Worldwide, diabetes (types 1 and 2) is a leading cause of death, associated with a 30% loss of life expectancy from diagnosis (Standl 2019). As of 2017, an estimated 425 million people have diabetes (types 1 and 2) worldwide (IDF 2017), of which T1D accounts for between 5 and 10% (You and Henneberg 2015). In the Netherlands, around 100,000 people have T1D (IDF 2017). A recent 25-year study reveals that the incidence of T1D is increasing by more than 3% per year in Europe (Patterson et al. 2019).

Self-managing diabetes involves, at heart, keeping one's blood glucose levels within as normal a range as possible, which is essential in order to reduce the chances of developing diabetes-related complications (e.g., neuropathy, nephropathy, heart and blood vessel disease, eye damage, or foot damage) and to live a good and productive life (Redman 2005). Knowing one's glucose levels—measuring and monitoring them—is vital to the process of managing them; well-informed decisions about insulin dosage require knowing not just the amount of carbohydrates one wishes to consume, but also the current state of one's blood glucose concentration.^2^ Glucose monitoring has developed much since early attempts at quantification in the mid-1800s, which led to the first development of urine glucose testing in 1908, and to the option, now standard practice, of testing blood glucose at home in the 1980s (Hirsch 2018).^3^ In 2004, the first continuous glucose monitoring (CGM) system was introduced, which measures glucose levels in real-time and which notifies users of potentially dangerous hypoglycemia (low blood sugar) or hyperglycemia (high blood sugar), thus "revolutioniz[ing] the way diabetes is managed, especially type 1 diabetes" (Hirsch 2018, p. 1).^4^

In fact, the Endocrine Society has recently recommended the use of CGM devices for adult patients with T1D in order to manage their blood glucose levels (Peters et al. 2016). CGM systems "use subcutaneous sensors to monitor blood glucose concentration, let patients track their blood glucose levels in near real time and adjust their insulin doses accordingly," and they usually have an alarm function that alerts people with T1D, and close others with whom they can share alarms, when blood glucose levels fall too low, rise too high, or rise or fall very quickly (McCarthy 2016, p. 1).

Data on how many people with T1D currently use CGM is scarce; one estimate is that in the U.S., where the technology was first developed, 15% of people with T1D used CGM in 2017 (Close and Brown 2017). This number is likely to have increased, but is still far from constituting a majority. In the Netherlands, CGM devices are not covered by insurance, and criteria for CGM provision to people with T1D by hospitals are currently very stringent. Only few people qualify, and qualification tends to be temporary. This means that most people, should they wish to use CGM, have to purchase it themselves, and costs are high enough to prohibit many, if not most, from being able to do so.^5^

In this paper, I argue that unequal access to CGM is potentially unjust in four ways, so that there are at least prima facie moral reasons for all people with T1D to have the option of managing their illness through CGM.^6^ The operative notion of justice throughout the paper, unless otherwise stated, is justice as fairness, where justice requires that "all societies meet healthcare needs fairly under reasonable resource constraints" (Daniels 2001, p. 9).^7^

First, in support of my claim, I review evidence that CGM use contributes substantially to the health and wellbeing of people with T1D, and argue that unjust health inequalities result when only some are able to use the technology.

Second, the restriction of CGM to a limited group of people not only relies on unjust socioeconomic inequalities, but it also produces unjust consequences. Drawing on Haverkamp et al. (2018), I argue that, to the extent that health is instrumental for relational justice, unequal access to CGM threatens relational justice through (1) unequal risks of stigmatization, (2) unequal risks of unemployment, and (3) unequal risks of enjoying a relatively equal number of pension years.

Third, I argue that CGM enhances the agency and autonomy of people with T1D. For instance, CGM use can help to increase control over one's illness and to ameliorate fears about one's blood glucose levels (especially fear of hypoglycemia), which is another area in which injustice occurs when not all people with T1D are able to use CGM.

Fourth, unequal access to CGM constitutes a specific form of epistemic injustice. In light of work by Carel and Kidd (2017), I argue that unequal access to CGM leads to unjust knowledge and experiential asymmetries between people with T1D who are and are not able to use CGM.

Finally, I outline current policy for CGM eligibility in the Netherlands in order to show that in this specific context, unequal access to CGM technology is, all things considered, unjust. In light of a number of recent studies that highlight the potential cost-effectiveness of CGM, I urge the Dutch government to cover CGM use for all people with T1D as part of basic health insurance.

## Health Outcomes and Health Inequality

To effectively manage T1D, it is imperative to keep blood glucose concentrations with a range that is as close to normal as possible; this means attempting to maintain euglycemia (normal blood glucose concentrations) by avoiding values that are either too low (hypoglycemia) or too high (hyperglycemia), so as to minimize potential complications. As Cryer (2014) describes it, selecting a glycemic goal for someone with T1D "is a compromise between the documented upside of glycemic control—the partial prevention or delay of microvascular complications—and the documented downside of glycemic control—the recurrent morbidity and potential mortality of iatrogenic hypoglycemia" (p. 2188). In plain terms, the more one aims at better glycemic control (to avoid future complications), the greater the chance of hypoglycemia (and the acute risks that it poses).

I want to focus, then, on these two crucial aspects of T1D management, which have been shown to be positively affected by CGM use: (1) glycemic control, and (2) hypoglycemia. The documented improvements by CGM within these two areas are a major reason behind the Endocrine Society's recent recommendation of CGM use for all patients with T1D (McCarthy 2016).^8^

First, CGM use has consistently been associated with better glycemic control (i.e., improved mean blood glucose levels over time).^9^ A number of studies have demonstrated that CGM use significantly improved glycemic control for people with T1D (e.g., DeSalvo and Buckingham 2013; Beck et al. 2017; Foster et al. 2016; Charleer et al. 2018). Using CGM was even found to significantly improve glycemic control for individuals with T1D who already achieved excellent control (Beck et al. 2009). A recent qualitative study also reveals how CGM use "can make living with T1DM safer and easier, while improving the time spent in the target glucose range," which, according to the authors, highlights "the immediate need for improved access and support for CGM" (Mohamed et al. 2019, p. 333). Good glycemic control is a crucial marker of health for people with T1D. It is not only associated with a reduced risk of diabetes-related complications, which can be debilitating and life-threatening (DCCT 2005), but it is also more generally associated with better quality of life (Jacobson 2004). Poor glycemic control is associated with negative outcomes like depression (Lustman et al. 2000), so that "[t]he probability of depression increases as glycemic control worsens" (Hassan et al. 2006, p. 526). That CGM use improves glycemic control, even for those already relatively well-adjusted, is therefore a highly valuable outcome that will have a significant impact on the health and overall quality of the lives of people with T1D.

Second, CGM has consistently been associated with fewer and shorter episodes of hypoglycemia.^10^ More must be said about this phenomenon, so that those especially who are unfamiliar with hypoglycemia may understand its significance for T1D management. The brain crucially depends on continual glucose supply in order to function—it is unable to synthesize or store glucose, its primary source of energy, and is therefore particularly vulnerable to glucose deprivation (Briscoe and Davis 2006). Hypoglycemia occurs when blood glucose concentrations fall below the glycemic threshold of 3.9 mmol/L or ~ 70 mg/dL,^11^ upon which "a sequence of responses is activated that includes release of neuroendocrine hormones (also called counterregulatory or anti-insulin hormones), stimulation of the autonomic nervous system (ANS), and production of neurogenic and neuroglycopenic symptoms to protect the brain and limit systematic effects of hypoglycemia" (Briscoe and Davis 2006, p. 115). Plainly put, in a self-preservative response, the body reacts to falling glucose levels by sending warning signals and attempting to counteract the process. Neurogenic symptoms (caused by falling glucose levels) include symptoms like shakiness, anxiety, sweating, palpitations, and hunger, while neuroglycopenic symptoms (caused by brain neuronal glucose deprivation) cause, among other things, confusion, difficulty in thinking and speaking, ataxia, seizures, coma, and finally, if untreated, death.

Hypoglycemia is "a fact of life" for people with T1D, who are estimated to suffer an average of two episodes of hypoglycemia per week, which runs into the thousands of episodes over a lifetime (Cryer 2010, p. 642). While early reports suggested that 2–4% of deaths of people with T1D are due to hypoglycemia, more recent reports indicate that this number is around 6–10% (Cryer 2010). In one study, self-reported severe hypoglycemia was associated with a 3.4-fold increased risk of death (McCoy et al. 2012). Aside from mortality, hypoglycemia also causes recurrent morbidity in most people with T1D (Cryer et al. 2003). What is especially problematic is that, "[w]hile the clinical presentation is often characteristic, particularly for the experienced individual with diabetes, the neurogenic and neuroglycopenic symptoms of hypoglycemia are nonspecific and relatively insensitive; therefore, many episodes are not recognized" (Cryer et al. 2003, p. 1902).

One recent review article of the effect of CGM use on hypoglycemia goes so far as to proclaim CGM a "solution" to hypoglycemia and the risk to suboptimal glycemic control that it poses (Adolfsson et al. 2018). Importantly, the sharing of predictive CGM information with family members, friends and/or caregivers "further reduces the risk of both moderate and severe hypoglycemic events" (Adolfsson et al. 2018, p. 55). While hypoglycemia is closely related to glycemic control (e.g., recurring episodes of hypoglycemia tend to indicate poor glycemic control), it is an important marker of T1D management in and of itself. Severe hypoglycemia, for one, predicts mortality in diabetes (Cryer 2002), and hypoglycemia more generally "impacts heavily on the well-being, productivity and quality of life of people with diabetes," so that "every effort should be made to minimize hypoglycemia while aiming for good glycemic control" (Davis et al. 2005, p. 1477). It must also be added that, aside from the adverse effects on the individual with T1D and those within their immediate surroundings, the economic burden of hypoglycemia is substantial (De Groot et al. 2018). Furthermore, in a real sense, CGM can be life-saving. Predictions of oncoming hypoglycemia can help a person act before they might otherwise lose the capacity to do so; and when a person experiences severe hypoglycemia while alone, the alarm function can warn others so that help can arrive on time.^12^ This is especially true for people who suffer from hypoglycemia unawareness (HU), which is an impaired awareness of the symptoms of hypoglycemia associated with a sixfold increased risk of hypoglycemia (Cryer 2010).^13^ Yet it also holds for those without HU, because, as mentioned before, many episodes of hypoglycemia are not recognized (Cryer et al. 2003).

Importantly, the use of CGM has been found to achieve its benefits "without imposing an additional burden on the patient or increased medical resource use" (Hommel et al. 2014, p. 845).^14^ Nevertheless, the costs of using CGM are currently prohibitive for many people. When only some people with T1D are able to benefit from the improvements to glycemic control and hypoglycemia associated with CGM use, and to make use of the invaluable alarm and alarm-sharing functions, then this will create and exacerbate health inequalities within a population that is already vulnerable.

One influential recent definition, which is apt for those with chronic illnesses like T1D, conceives of health as "the ability to adapt and to self-manage" (Huber et al. 2011, p. 2). To the extent that CGM use helps people with T1D do exactly that—adapt and self-manage (with the CGM information and alarm function)—their very health is improved. Those who are denied this option will be left behind and, accordingly, have no chance at having their health enhanced. These considerations provide at least a prima facie reason for universal access to CGM for people with T1D. Distributive justice, at its core, concerns "fair, equitable, and appropriate distribution of benefits and burdens" (Beauchamp and Childress 2013, p. 250). No one with T1D has chosen their condition; all bear its burdens. That only some people with T1D should receive the benefits associated with CGM, thus giving them (but not others) a better chance at controlling their blood glucose levels, their illness, and their lives, hardly seems fair.

## Health Inequality and Unjust Consequences

I have shown that CGM devices are associated with a number of important health benefits for people with T1D, and I have argued that inequality in access to these devices is unjust to the extent that they create and worsen health inequalities as well as other qualitative aspects of the lives of people with T1D. Yet, there is more to it than this.

Health not only has intrinsic value; it is also instrumentally important. There are many accounts of the instrumental value of health; that it has special moral significance for fair equality of opportunity (Daniels 2008), for instance, or that it is essential for living a decent human life (Venkatapuram 2011). I want to restrict my argument here to the potentially negative effects of health inequalities on relational equality, or on what is "necessary for functioning as an equal citizen in a democratic state" (Anderson 1999, p. 316). As Haverkamp et al. (2018) argue, not only the causes, but also the consequences of health inequalities are important in understanding why these are unjust. They propose that "equality in health is instrumental for justice," and that "socioeconomic inequalities are unjust because they lead to relational injustices" (Haverkamp et al. 2018, p. 312). More specifically, they argue that health inequalities threaten the ideal of relational equality in three ways: (1) through unequal risks of stigmatization, (2) through unequal risks of unemployment, and (3) through unequal chances of enjoying a (relatively) equal number of pension years (Haverkamp et al. 2018, p. 312).

What does this mean for justice in the case of unequal access to CGM devices for people with T1D? First, it must be noted that socioeconomic inequality initially creates the problem of unequal access: if all people with T1D were well-off enough to afford CGM, then there would be no inequality to speak of. Perhaps not everyone would decide to buy them, but that is not a matter of justice. Second, one must ask how this socioeconomic inequality, expressed through the unequal ability to afford potentially highly beneficial CGM systems, can lead to relational inequality and thereby constitute a form of injustice.

First, there is the matter of unequal risk of stigmatization. People with T1D experience a significant amount of stigmatization in many areas of life (Jaacks et al. 2015; Balfe et al. 2013). One study found that other people's perceptions of diabetes (i.e., diabetes stigma) had significant negative effects on the emotional lives of people with T1D and T2D (e.g., resulting in experiences of guilt, blame, shame, embarrassment, and isolation), as well as on their social lives and their diabetes management; the impact of diabetes stigma on all aspects of their lives was significantly associated with a higher HbA1c (Liu et al. 2017). Another study of young women with T1D found that they "were not able to control the negative unexpected responses of other people to their diabetes, which made them feel emotionally vulnerable, especially in the context of family and school communities and when people exhibited misconceptions and lack of understanding diabetes" (Rasmussen et al. 2007, p. 303). A review by Schabert et al. (2013) found that people without T1D assume that diabetes is not a stigmatized condition, while those with T1D report that stigma is a significant concern for them and is experienced across a number of life domains, for example in the workplace and in personal relationships.

One major benefit of CGM is that it can be operated on one's phone, which is, for better or worse, a widely socially accepted device. People with T1D can keep track of their glucose levels clandestinely by simply checking their phone screen. To the extent that stigmatization is associated with visibility of illness, the fact that one may very easily keep glucose checking private is an important means toward decreasing potential stigmatization.^15^ It goes deeper than this, however. The fact that CGM use, as I have shown, reduces hypoglycemia and improves glycemic control, has far-reaching consequences for stigmatization. Socially identifiable characteristics pertaining to T1D are an important contributor to the experience of stigma (Liu et al. 2017). Experiencing hypoglycemia in public can be a major source of fear (Rasmussen et al. 2007)—both for its own sake, and for the perceived stigma. The experience of stigma, moreover, has been associated with higher HbA1c—that is, with lower glycemic control—and poorer self-reported glucose control (Liu et al. 2017). There are grounds to assume, therefore, that CGM is likely to contribute to decreased stigma within these important areas. As such, when only some people are able to use CGM, this puts those who cannot at an equal risk for stigmatization compared to those who can.^16^

Second, there is the matter of unequal risks of unemployment. Again, the power of CGM to reduce hypoglycemia and to improve glycemic control is crucial. Even non-severe episodes of hypoglycemia have a substantial impact on work productivity (Brod et al. 2011). It stands to reason that people with T1D who experience significantly fewer episodes of hypoglycemia and better glycemic control (that is, those who are by these standards healthier and better adjusted) are less likely to miss work or to experience interruptive hypoglycemia or hyperglycemia at work. Furthermore, the association of poor glycemic control with psychiatric conditions like depression (e.g., Northam et al. 2004) has serious consequences in this area, because mental illness is associated with significantly higher rates of unemployment compared to the general population (Baron and Salzer 2002). There is reason to expect, then, that those people with T1D who are not helped by CGM (compared to those who are) are at greater risk of unemployment.

Third, there is the matter of unequal chances of enjoying a relatively equal number of pension years. In the more extreme scenarios, CGM use can prevent death from severe hypoglycemia. This, by definition, means a gain in life—potential pension years included—that is denied to those who cannot use CGM. Furthermore, given that CGM use is associated with better glycemic control, this means a significant decrease in the chances of developing diabetes-related complications, which seriously threaten the health and quality of life of people with T1D later in life—including the pension years (see, e.g., Huang et al. 2007). Blindness, kidney failure, and chronic foot ulcers will do much to curtail if not prevent the enjoyment of one's pension years. This third consideration appears to be the weightiest; it is probably associated with the greatest, and most unjust, differences between the states of people whose health is positively affected by CGM use and those whose health is not.

CGM use, therefore, not only contributes to unjust health inequalities, but it also threatens relational justice by producing unequal risks of stigmatization and unemployment, as well as unequal chances of enjoying a relatively equal number of pension years.

Having discussed the effects of CGM on health inequalities and their consequences for relational justice, I will now address how agency and autonomy may be affected in ways that lead to injustice.

## Agency and Autonomy

There is a large philosophical literature on the concept of agency; its use in bioethics has recently received increased attention, while its definition and meaning have been the subject of contention (Barreda et al. 2016). To avoid being bogged down by details that are irrelevant to the purpose of this paper, I will understand agency broadly in relation to what it entails for people with T1D. In general terms, an agent is "a being with the capacity to act," and agency "denotes the exercise or manifestation of this capacity" (Schlosser 2015, p. 1). Within the sphere of the life of a person with T1D, a crucial area in which to exercise one's agency is the management of illness. This, among other things, means regulating one's insulin intake and ensuring that one's blood glucose levels remain within a safe range. In order to do so, one must act, often many times a day—adjusting insulin doses, ingesting carbohydrates when necessary, and so on. Now, CGM is probably superior in aiding many of these processes; the easier it is to check one's blood glucose levels, the better. The real boon to agency, however, is provided by the trend and alarm functions. These offer highly meaningful insight into one's condition and into *how one should act*. They act as guides to practical action; they are intended as such. When the alarm of his CGM device sounds, and John sees that his blood glucose levels are quickly falling to a potentially dangerous low value, there is little in his life within that moment that is as relevant and as significant to him. He needs to take practical action. Of course, in order to properly exercise his agency, John must respond; he must also have a means of acting upon the situation as sketched by the CGM. Yet, the initial call to action is critical in pushing along the entire process in which John exercises his agency within the context of (responding to) the demands of his illness. Now, if one always received these calls to action through other means (if, for instance, the body's own alarm system of sensations were perfect), then CGM might be less important to agency, or even not at all. But, crucially, there may be many reasons why a person does not experience changes in blood glucose levels upon which one nevertheless should act. The most obvious case is provided by people who suffer from HU—these people do not experience the “classic” symptoms of hypoglycemia, which can lead to life-threatening situations (Gerich et al. 1991; Bakatselos 2011). Yet, between the extreme case of HU, and the ideal of perfect glycemic awareness, lies a range of imperfect experiences. Hypoglycemia is not always obvious (Kenny 2014). There are many factors that may mask or delay awareness of blood glucose concentrations on which one ought to act: numerous distractions, strong emotions, tiredness, and so on. In this way, then, CGM is an important way of supporting and enhancing agency, especially in cases where, for whatever reason, symptoms of action-demanding (changes in) blood glucose levels are imperfectly perceived.

There are two further ways in which agency may be enhanced by CGM. First, the data provided by the sensor offers a range of statistics about recorded blood glucose levels. Some of this data can be learned from the device at a moment's glance; for more extensive insight, data can typically be downloaded and mapped onto a computer. Trends and means can be charted so as to receive a bird's eye view of how one's blood glucose levels were over a selected period of time, sometimes even months.^17^ This creates an additional means of making decisions for the person with T1D, an important basis for practical rationality. For instance, if blood glucose levels are shown by CGM data to be consistently high over a period of weeks for one specific part of the day, then a person may (or better yet, should) consider taking more insulin with their meal for that part of the day. This is the kind of insight that only comes from exposure to this kind of data. Additionally, this information can be shared with health care providers, so that, together with patients, they may examine and interpret what it means for treatment and self-management of T1D.

Second, a hugely valuable feature of most CGM devices is that one can share the real-time data with other people. Through an app, Rachel with T1D can share her glucose readings with her brother Mark, who will then be notified (based on parameters that can often be personalized) when there is a potentially dangerous blood glucose trend. If Rachel happens to be home alone when she experiences a severe episode of hypoglycemia means that Mark will be notified and can inform emergency services, potentially saving his sister's life. There are situations in which this form of sharing must not only be a great general source of comfort for one with T1D and for those who care about them, but where it can also be potentially life-saving. Even in less severe cases, the alarm can aid early detection of falling glucose levels and thus prevent severe hypoglycemia and/or decrease time spent in hypoglycemia. When someone with T1D experiences hypoglycemia, their agency is minimized through the symptoms it causes (especially neuroglycopenic symptoms like confusion, difficulty in thinking and speaking, or ataxia), and even in some cases completely undermined (as in when one loses consciousness). The ability of partially offloading agency to (1) the CGM itself, and (2) others via CGM alarms is an invaluable way of, ultimately, reinforcing one's own agency and preventing it from being compromised.

Personal autonomy is the ability for agents to govern themselves and to give shape to their own lives (Buss and Westlund 2018). Agency is important for autonomy, to the extent that the first contributes to the latter. I want to use a more specific conception of autonomy; namely, one that conceives of autonomous action as choices and decisions that, under non-ideal conditions, are made (1) intentionally, (2) with understanding, and (3) without controlling influences that determine the action (Beauchamp and Childress 2013, p. 104). While the first two conditions are likely to be relevant here,^18^ I want to focus on the third, because I think it is the most urgent. In any case, if one of the three conditions for autonomy is undermined (or significantly enhanced), this is enough to make a judgment about (the effects on) autonomy as such.

The condition of noncontrol holds that "a person be free of controls exerted either by external sources or by *internal states that rob the person of self-directedness*"^19^ (Beauchamp and Childress 2013, p. 104). One of the internal threats to autonomy that Beauchamp and Childress propose is mental illness, which disproportionately affects people with T1D and which can occur at all stages of the illness, from diagnosis onward (Levy 2016). Now, it is unlikely that most mental illnesses will be affected by CGM use. Eating disorders, major depression, and a myriad other psychiatric conditions are unlikely to be bettered by how one is able to track one's blood glucose levels. Yet, there are both clinical and sub-clinical psychological disturbances related to T1D that *are* likely to undermine autonomy in the noncontrol sense, and that *are* likely to be helped by CGM use. I will focus on one such phenomenon, namely, fear of hypoglycemia (FOH), which is a pervasive problem among people with T1D. FOH—the fear of experiencing a hypoglycemic episode—can be debilitating, and is detrimental to diabetes self-management and psychological wellbeing (Cox et al. 1987; Martyn-Nemeth et al. 2015). This fear creates "a major barrier in achieving glycemic control and good quality of life" (Schmidt 2017, p. 1360). In fact, one group of researchers has argued that managing hypoglycemia may actually be more about fear management than about glucose management (Vallis et al. 2014). This makes sense when one thinks about what T1D means: since insulin is no longer or too little produced by the pancreas, one has to take over this function by introducing extraneous insulin, which means that one can take too much of it. The consequences of taking too much are, as previously discussed, life-threatening. It is no wonder, then, that a significant number of people with T1D are fearful of having a hypoglycemic episode. Although clinical prevalence of FOH is estimated at 10–5% of the population of people with T1D (Schmidt 2017), there is reason to think that sub-clinical fear is even more widespread. Qualitative research reveals that fighting fear in the search for safety is part of the essential structure of mastering diabetes (Ingadottir and Halldorsdottir 2008). Anecdotal evidence from stories on any of the many forums on the Internet dedicated to T1D further suggests that one major source of fear for people with T1D is nighttime hypoglycemia. This fear is not ungrounded, for many people with T1D *do* experience hypoglycemia during the night, as at potentially any other time of day. Being afraid to go to sleep (and thus sleeping too late), setting alarms during the night (and thus not sleeping well), and keeping ones levels too high so as not to risk them running low are consequences of this fear that, in turn, significantly negatively affect the wellbeing of the people who face it.^20^ FOH, therefore, undermines autonomy in the noncontrol sense. I assume that all people with T1D want to sleep well, want to experience wellbeing, and do not want fear to drive their actions—especially if these are ultimately detrimental to them (for instance, if they impede good glycemic control). Fear undermines one's ability to govern oneself well. As should hopefully be clear by now, CGM is an important means of at least alleviating some of this fear, and bolstering (or even restoring, in the more severe cases) the noncontrol condition of autonomy. Knowing that an alarm will wake one up, and/or that a loved one will receive an alarm should a dangerous situation develop, can be a major source of reassurance.^21^ Whether it is enough to assuage clinical symptoms of FOH, I do not know (more research is needed here), but I am convinced that it can do a great deal of good.

One might accept this, yet still argue that only people whose autonomy is undermined in the ways that I have described should have access to CGM. I would argue, in turn, that the person with T1D whose autonomy (at least in the control sense) is never negatively affected does not practically exist. Qualitative research shows that autonomy is essential to mastering diabetes (Ingadottir and Halldorsdottir 2008). True, autonomy may be more greatly enhanced (or more dramatically restored) for some than for others. Nonetheless, since CGM is likely to enhance autonomy across the board, for all persons with T1D, it stands to reason that all should at least have the option of trying it, and if it helps them, of using it.

Having autonomy over one's life and being able to exercise one's agency is important for people generally, and particularly when it comes to self-care for people with chronic illnesses like diabetes (e.g., Sousa et al. 2008; Naik et al. 2009). If the two are significantly enhanced by CGM for people with T1D, whose lives have already become difficult to manage due to their illness, then it would appear to be unjust for only some to have access. Not only that, but respect for autonomy is a cornerstone of bioethical theory—perhaps even the single most important principle (Gillon 2003). Giving people with T1D the option of managing their illness through CGM is in itself a very important way of respecting their autonomy.

## Epistemic Injustice

I want to discuss a final sense in which not having access to CGM devices can constitute a form of injustice, in light of what Fricker (2007) has called epistemic injustice. At its core, this distinctively epistemic form of injustice entails "a wrong done to someone specifically in their capacity as a knower" (Fricker 2007, p. 1). I understand the concept more broadly than how it was initially used by Fricker (2007), thus taking seriously Pohlhaus's (2017) suggestion that the concept be used more open-endedly in order to allow discussion in other and more diverse fields. Havi Carel and Ian James Kidd, building upon Fricker's groundwork, have argued that ill persons may be especially vulnerable to epistemic injustice (Carel and Kidd 2014). They have also explored some of the forms that epistemic injustice may take within the practices of medicine and health care (Kidd and Carel 2017; Carel and Kidd 2017). I want to focus in particular on two kinds of asymmetry—knowledge and experiential—that are outlined by Carel and Kidd (2017). While they discuss these asymmetries in the context of the structural features of healthcare systems, primarily between those who wield medical power and patients, I want to examine them instead within the context of relations *between patients*. That is, rather than characterize asymmetries vertically, for instance between a doctor and patient, I want to conceive them horizontally—among people with T1D. More specifically, I argue that both asymmetries will occur between those who use CGM devices from those who are unable to, and that this is unjust.

Before continuing, more needs to be said about the experience of patients with T1D, and about the significance of blood glucose information. Being diagnosed with T1D changes one's relationship with one's body. A healthy person may never, or only very rarely, think about what their blood glucose levels are or what their being a certain way might mean; this information does not figure in their practical decision-making (other than, perhaps, when a feeling of hunger is linked with low blood sugar).^22^ On the other hand, a person with T1D who seeks to manage their illness is, if not constantly, at least very often concerned with their blood glucose levels.^23^ I deliberately choose the word 'concerned' to reflect both a neutral kind of invested interest, as well as a more emotional form of *concern*. What are one's levels when getting ready for bed? Too low, and a potentially dangerous episode of nighttime hypoglycemia might ensue. Too high, and an unpleasant episode of nighttime hyperglycemia might arise, possibly making it more difficult to guide one's levels within a normal range the following day. It is worth emphasizing here what Carel and Kidd (2017) write about the experience of chronically ill persons, who "experience their illness not as localized biological dysfunction, but as *ongoing*, *pervasive*, perhaps *all-encompassing*—a definitive 'mode of being,'" and, "although illness may be only one aspect of that 'mode' or way of life, it can come to dominate their identity either as they conceive it, or, more significantly, as others do" (p. 336).^24^ The extent to which people with T1D experience their illness as a *mode of being* is likely to vary among individuals; but that their illness is omnipresent is unquestionable. One cannot but be reminded of one's illness when one has to inject (or, alternatively, “pump”) insulin throughout the day, measure one's blood glucose levels multiple times a day, and so on.^25^ One may handle it all very well, but one cannot forget. The reminders are too frequent and too powerful—and when one is called on by these necessities of managing one's illness, all other activity is interrupted, if only for a moment.

Blood glucose information is the cornerstone of successful diabetes management; it is practically highly useful, for instance in making a decision about what dose of insulin to take. Blood glucose information can also be more theoretically significant, in the sense that aggregates of data, or trends, can provide insight into and aid one's management over time. These more pragmatic considerations also become layered with personal significance for people with T1D. The information that blood glucose concentrations provide to the person with T1D is a highly intimate form of information that is personally meaningful. To a person with T1D, few other forms of information will affect their lives as deeply as the amounts of glucose in their bloodstream.^26^ Not only does this information guide practical decision-making, but it can also help to explain events and personal experiences, for instance how one's mood may have been affected by one's illness,^27^ or the effects of certain forms of activity, food, or drink, on one's body. This provides an opportunity for learning; to know what makes one feel good or poorly is to know what to avoid or approach and vice versa, mediated by information about effects on one's body.

Ideally, one would have perfect knowledge of one's blood glucose levels at any given moment; a kind of one to one relation of the actual state of one's body to information about that state. Additionally, and again ideally, one would have perfect access to knowledge of one's blood glucose trends at any given moment when such trends are meaningful for practical action (that is, when this would lead to taking a beneficial action, such as consuming glucose or taking insulin). Before a cure emerges, we might yet approach this ideal through technological advances. For now, however, CGM already exists; and it brings us as close to this ideal as ever we have been before.

The near-real-time monitoring of blood glucose concentrations provides important and matchless knowledge. The alarm feature (that is, the ability of the device to alarm its users to potentially dangerous states of affairs), is also a way of knowing distinct from the monitoring itself. Crucially, the alarm can provide knowledge before the wearer of the CGM device might otherwise become aware of the situation. Consider this example. A person with T1D is about to give a presentation. They checked their levels not long ago and feel fine. *As far as they know*, at that moment, there is no reason to take any sort of action. Now, picture this same person in exactly the same situation, but with CGM. As they are about to give the presentation, all the conditions just described hold—but they receive an alarm that their blood glucose levels are going to be low in twenty minutes. Assuming that the CGM reading is accurate, and assuming also that both their blood glucose measurement earlier was accurate as well as their feeling-fine (i.e., they do not feel any symptoms of hypoglycemia), then the CGM offers them an additional, a unique, and, most importantly, a *better* way of knowing the state of affairs regarding their condition. It gives them a *reason* to undertake practical action—suggesting, moreover, not just *that* they act, but also *how* to act (i.e., by increasing the amount of glucose in their bloodstream). Practically speaking, they would do best in this moment to ingest glucose in some form.^28^ I think that the situation just sketched is not far-fetched; in fact, I think it a common experience when using a CGM device. Two of the main advertised features of the CGM device are, after all, (1) to monitor, and (2) to predict blood glucose levels. As such, it provides a distinct way of knowing that is not reducible to other forms of blood glucose measurement. This, then, is what is withheld from people who are not able to make use of CGM devices, whose capacity to know and to make use of the knowledge provided by the CGM device must nonetheless be equal to those who do get to use it.^29^

Every person with T1D is a knower in their own right and should be respected as a knower. As I have argued, one of the most fundamental and intimate sources of knowledge for a person with T1D is information about blood glucose. It is a uniquely meaningful source of knowledge not shared by those without the particular illness, which is furthermore essential to the continued self-care and overall health of people with T1D. As such, people with T1D should be able to access their blood glucose information, that most significant, yet otherwise invisible indicator of the state of their body and health. One might object that this argument can be applied to any form of knowledge that is in some way important for practical action. For instance, if everyone could (in principle) have their genome sequenced in order to possess the best possible knowledge about potential genetic disorders that they might someday develop, does this entail that everyone has a positive right to such knowledge?^30^ This is a difficult question, but an important one in light of new technologies that afford enhanced or even entirely novel forms of knowledge about the state of one's bodily functioning, health, and so on. Whether questions of epistemic justice will arise is bound to depend at least in part on the case in question (e.g., the technology and context) and will likely include grey areas. I would argue that, if the knowledge to be gained is clearly important for the direction that a human life might take, if it is not only important as an abstract kind of knowledge but if it has persistent, real-life implications for one's life, and if, finally, such knowledge has at least the potential but especially if it can reasonably and convincingly be shown to lead to a better life (e.g., significantly better physical and/or mental health outcomes), then this constitutes at least a prima facie reason to consider providing such knowledge to everyone. This seems to me to be the case for people with T1D and the knowledge that CGM provides.

Carel and Kidd (2017) distinguish two kinds of asymmetry in their discussion of epistemic injustice in healthcare. The first is a potential *knowledge* asymmetry, which occurs when certain forms of knowledge are privileged over others, while the second is a potential *experiential* asymmetry, which occurs when certain forms of experience are privileged over others. As I have described, the use of CGM constitutes a significant quantitative change in the life of a person with T1D over previous forms of glucose measurement: the way of knowing (the details of) one's illness is changed, providing a more complete image of how one's body functions. It goes further than this, however, for the very experience of that illness is also changed. Knowing that a CGM device is running in the background, recording your blood glucose levels and, potentially, warning you if anything is amiss, is an experience quite radically new for a person with T1D who has not used CGM before. No longer having to take blood from your fingers,^31^ but having only to look at your phone for a glucose status update, changes how one goes about in the world.

What consequences does this have for the discussion of who should be able to use CGM devices? The upshot is that, if only some people with T1D are granted access (through insurance or private means) to the ways of knowing and the unique experience that CGM devices provide, then this constitutes a form of epistemic injustice to those who are denied such access. When only some are able to use CGM, but not others, then this creates and perpetuates unjust knowledge and experiential asymmetries between the two groups. CGM provides not just a crucial aid in the management of T1D, but it is also a distinct way of knowing one's blood glucose levels—not just in the moment, but also how they change and react to various stimuli over time. And, for the person with T1D, knowing these things is not just an abstract tool for managing their illness (which is already something). It is also a unique way of knowing their body; a way of being in the world, which no person with T1D should have withheld from them. Qualitative research underscores the importance of knowledge, understanding, and experience in relation to diabetes (Ingadottir and Halldorsdottir 2008). That some people should be barred from the forms of knowledge and experience that CGM provide creates and perpetuates epistemic injustice. Of course, people with T1D should not be forced to use CGM. There may be valid reasons why a person would not or no longer wish to use make use of the technology. It is the fact that many people currently cannot choose their way of knowing and experiencing their illness that appears to be unfair.

To get back to the definition of epistemic injustice as a wrong done to someone as a knower, one has to ask *who* is doing the wrong in the case of CGM devices being provided to only some people with T1D but not others. For this, we have to know the context—the conditions and policies under which the relevant real-life decisions are made under conditions of scarcity. In the next section, I discuss the concrete case of CGM provision in the Netherlands.

## Current Policy: The Case of the Netherlands

The upshot of my argument so far is that there are prima facie reasons of justice for all people with T1D to be able to use CGM. Of course, whether people with T1D ought to be provided with CGM, all things considered, will depend on the more specific context. In this section, I will discuss one such context, namely, the health care system in the Netherlands, to argue that the cost of CGM for people with T1D in the Netherlands ought to be covered by basic insurance.^32^

Currently, whether or not a patient is eligible for CGM coverage in the Netherlands is stipulated by Zorgverzekeraars Nederland (ZN), which is an umbrella organization comprising ten Dutch health insurers.^33^ There are several criteria for whether or not a person is eligible for CGM. Specifically, one must fulfil at least one of the following criteria^34^:

- Children < 18 years old with type 1 diabetes.
- Adults with poorly managed type 1 diabetes (despite standard controls, blood glucose levels remaining high with an HbA1c of > 8% or > 64 mmol/mol).
- Pregnant women with established diabetes (type 1 and 2).
- Women with a pregnancy wish with gestational diabetes (type 1 and 2).
- Patients with type 1 diabetes, who suffer serious episodes of hypoglycemia and/or are unable to detect hypoglycemia (i.e., are hypo-unaware).

If none of these criteria apply, then one is ineligible to receive CGM from the hospital. The decision regarding CGM, according to the document specifying the criteria, "should be best suited to the individual care requirements of the patient."^35^

Although one may and probably should question some of the requirements,^36^ the real problem extends beyond the specific eligibility criteria. The crux is that funding for CGM currently derives from individual hospital budgets; hospitals have to negotiate independently with health insurance companies (and justify their requests to them) about coverage for CGM. This means, practically, that there is only a limited budget available for health care practitioners at hospitals to offer patients CGM. It also means that some hospitals cannot offer CGM at all, due to the restrictions and preferences for more “experienced” centers by insurance companies.^37^

Every medical specialist can prescribe CGM to a patient, but ultimately the team of practitioners at one's hospital decides whether or not they are eligible for hospital provision. While practitioners may seem to have some freedom in light of the eligibility criteria, the small hospital budgets for CGM provision practically means that only very few patients who would benefit from CGM (which, as I have shown, are virtually all people with T1D) are offered hospital use of CGM. Moreover, within the current arrangement, CGM provision tends to be temporary; it can be stopped, and likely will be, as soon as a patient no longer meets the relevant criteria.

An additional argument for providing all people with T1D with the option of using CGM is also revealed here. With full coverage, health care practitioners would no longer have to make difficult decisions regarding whether or not patients will receive CGM. Assuming that health care practitioners know the benefits of CGM, and that they have the interests of their patients at heart, it can be taxing to have to deny patients such use—not on the grounds that patients would not be helped by CGM, but simply because there is insufficient funding.

On the whole, then, allowing all people with T1D access to CGM seems like the right thing to do. It is what justice appears to require, and will very likely bring about the most good for people with T1D, for those people who care about them, and for those people who are charged with providing care for them. In an ideal world, everyone would have full access to all the health care services and medical interventions that they need. The reality is, of course, that costs must be weighed against benefits when it comes to the allocation of scarce health care resources. The ideal being unrealizable, it must at times be checked; and focus should be placed on prioritization, which is a difficult task indeed (Gordijn and Have 2011). It does not help that primary care aimed at helping people with chronic illnesses appears to be in decline (Mayes and Armistead 2013).

The main consideration against implementing comprehensive access to CGM appears to be the cost. Yet, a number of studies have assessed the cost-effectiveness of CGM for people with T1D and have found that real-world use of CGM devices for people with T1D can be cost-effective. In the United States, McQueen et al. (2011) found that, for adults with T1D, who use multiple daily insulin injections, CGM was associated with an incremental cost-effectiveness ratio (ICER)^38^ of approximately $45,033 [€40,917]^39^ per quality-adjusted life year (QALY); Huang et al. (2010) found that the ICER of CGM use was $78,943 [€72,635] per QALY; and Wan et al. (2018) established a higher ICER of $98,108 [€89,140] per QALY for lifetime use of CGM—noting that, by extending sensor use from 7 to 10 days, the ICER was reduced to $33,459 [€30,327] per QALY. This research reveals an "important expanded view on the contemporary economic value of CGM in T1D" (Wan et al. 2018, p. 7) and confirms the value of CGM for these patients while adding economic viability and cost-effectiveness as additional arguments to the relevant moral considerations. A Canadian study by Chaugule and Graham (2017) found CGM to be cost-effective for people with T1D who use multiple daily injections at an ICER of $33,789 [€22,858] per QALY. A recent study in Sweden found that CGM use yielded an ICER of SEK 164,236 [€14,410] per QALY gained (Jendle et al. 2019), while a study in the United Kingdom found that CGM use led to an ICER of £12,233 [€14,279] per QALY (Roze et al. 2016). It must be noted that the latter two studies examined CGM use in combination with continuous subcutaneous insulin infusion (i.e., combined with the use of an insulin pump). To date, one study has assessed the cost-effectiveness of CGM from a societal perspective specifically in the Netherlands. VanGenugten (2010) compared self-monitoring of blood glucose (SMBG) to CGM use in people with T1D and found that, overall, compared to the SMBG group, 0.463 QALYs were gained in the CGM group, with a total cost per QALY gained of €21,731 (VanGenugten 2010). It must be remembered that the prevention of hypoglycemia (and better glycemic control) associated with CGM use reduces not only the burden for individual patients, but also the economic burden for society (De Groot et al. 2018).

What does this mean for Dutch health care policy? In the Netherlands, health insurance is compulsory. The Dutch Care Institute (Zorginstituut) advises the Minister about what should be included in the basic insurance package according to four criteria: necessity, effectiveness, cost-effectiveness, and practicability (Zorginstituut Nederland 2018). An important consideration for decisions about what to include in the basic package is the relevant disease burden, which has to be weighed against the cost-effectiveness of a potential medicine or intervention—the higher the disease burden, the higher the willingness to pay for health gains (QALYs). The lowest category of disease burden, for instance, is associated with a willingness to pay of €20,000/QALY, while for diseases in the highest category this can go up to €80,000/QALY (Zorginstituut Nederland 2018). These thresholds are not absolute; they are meant as guides to policy and, as such, may be subject to debate.^40^

The disease burden of diabetes is currently ranked third in Disability Adjusted Life Years (DALYs) by the Netherlands National Institute for Public Health and the Environment (2020a).^41^ The disease burden of diabetes in 2015 (latest figure) was given a weight factor of 0.177,^42^ which places it in the lowest category^43^ of a maximum additional cost of up to €20.000/QALY to be considered for inclusion in the basic insurance package (Zorginstituut Nederland 2018). The study by VanGenugten (2010) of CGM cost-effectiveness in the Netherlands places it only slightly above the maximum willingness to pay threshold (+ €1731), as would the estimate from the Canadian study (+ €2858) by Chaugule and Graham (2017). The estimates from the Swedish and U.K. studies place the additional cost per QALY well below the maximum additional cost threshold (− €5590 and − €5721, respectively). The relatively high ICER estimate by Wan et al. (2018) appears to be somewhat of an outlier, although the ICER estimate with extended sensor use of €30,327 per QALY is closer to the estimates established in other studies.^44^

While more research into the cost-effectiveness of CGM in the Netherlands would be helpful, as it stands CGM coverage for people with T1D is very close to meeting the maximum additional cost per QALY requirement for the technology to be taken up as part of basic insurance coverage. An additional point to consider is that CGM technology is becoming cheaper over time (Neinstein 2019), which is likely to further decrease ICER estimates accordingly. With regard to the other criteria set forth by the Dutch Care Institute, I hope to have shown that CGM scores high on both the effectiveness and necessity criteria. As for the final criterion of practicability, although (as far as I know) there are currently no feasibility studies about implementing CGM in the Netherlands, the fact that CGM is already provided to groups of people at hospitals suggests that it would be only a relatively small adjustment to expand the system to include all people with T1D.

In light of the specific, context-sensitive considerations about cost-effectiveness in addition to the more general moral concerns about justice that I have discussed, it is my contention that the Dutch government ought to direct health insurers in the Netherlands to include CGM in the basic insurance package for people with T1D. Current insurance policy in the Netherlands fails to meet the demands of justice and ought to be changed. At least in the Netherlands, all people with T1D should have access to CGM.

## Conclusion

I have argued that there are a number of prima facie moral reasons for all people with T1D to have access to CGM as a matter of justice. More precisely, I argued that unequal access to CGM for people with T1D results in unjust health inequalities, threatens relational justice, creates and exacerbates injustice in the exercise of agency and autonomy, and leads to epistemic injustice. I discussed current CGM policy in the Netherlands, whose stringent criteria and limited funding only provide coverage for a selected few, and I have argued that this policy must be changed, especially in light of the potential cost-effectiveness of basic CGM coverage. Justice would require that access to CGM be covered by basic insurance for people with T1D.

The justice-based reasons for providing CGM do not, of course, entail that CGM ought to be provided to people with T1D everywhere at any cost. They are moral reasons that ought to be seriously considered, even if they may ultimately be outweighed by other considerations. In places where very basic healthcare needs cannot be met, for instance, one would hardly argue that people nevertheless ought to be provided with CGM. In a country like the Netherlands, where CGM can potentially be provided, especially at a reasonable level of cost-effectiveness, the case for offering CGM to all people with T1D is much stronger and more acute. I have focused on the case of the Netherlands, but my arguments provide at least prima facie moral reasons for CGM coverage for people with T1D in other countries, too. Given the speed with which diabetes-related technology is advancing (e.g., a next generation of implantable real-time CGM is already on the market), a discussion of distributive justice in this area is both timely and pressing.

## Footnotes

1 Insulin is usually administered via multiple daily injections (MDI) or continuous subcutaneous insulin infusion (CSII), also known as an insulin pump. I keep my account of T1D simple and minimize medical terminology, here and elsewhere, for the purpose of this paper.

2 Among other things. Other factors, like recent or expected physical exercise, are also relevant.

3 Before this, one had to travel to a physician's office in order to have one's glucose levels measured.

4 I was diagnosed with T1D almost 10 years ago, and am therefore intimately familiar with the disease. I am fortunate enough to be able to use CGM. In this paper, I draw upon personal experience where this is helpful. I have no affiliation with any company producing CGM technology. Having said that, part of the motivation for writing this paper was the realization that came after personal CGM use, namely, that no person with T1D should be denied this option.

5 A CGM system consists of a subcutaneous sensor, a transmitter, and a device from which to read the transmitted information (which can also be a mobile phone). Sensors and transmitters have to be regularly changed, though the latter less frequently. By my own calculations based on current prices in the Netherlands, it costs around €330 per month or nearly €4000 per year to use the latest Dexcom G6 CGM system. At current rates in the Netherlands, this constitutes ~ 11% of modal average monthly income, or ~ 34% of monthly income for people on living wage (for income data, see Trading Economics 2019).

6 Many of my arguments may also apply to people with type 2 diabetes (T2D). In fact, I think they do. Nevertheless, I focus on T1D because (1) the evidence for beneficial effects of CGM use for T2D is not as robust as for T1D (Wood et al. 2018), (2) while T2D may be reversed, e.g., through diet and exercise, T1D—until a cure emerges—is inevitably a lifelong illness, and (3) I thereby avoid moral questions about individual responsibility for illness.

7 I focus on considerations of justice, but there may also be other moral considerations in favor of extending CGM access to all people with T1D (e.g., one might argue to this end from a principle of solidarity).

8 It must be noted that another form of glucose monitoring, flash glucose monitoring (FGM), has also been developed (i.e., Freestlye Libre). It works with a sensor, like CGM, but it does not provide real-time information (one must swipe to retrieve data). It, therefore, has no alarm or alarm-sharing function. While people with T1D stand to gain much by FGM compared to traditional finger prick blood tests, the most significant benefits will accrue from CGM use, particularly through the alarm function. CGM has been shown to outperform FGM in key areas like time spent in hypoglycemia (Reddy et al. 2018).

9 This is usually measured by comparing Hemoglobin A1c (HbA1c) test results over time. Without venturing into technical detail, HbA1c testing provides a rough indicator of how much glucose was in the blood on average during a period of time before testing (usually up to three months prior), and thus of how well one has kept blood glucose levels within a target range.

10 For the sake of simplicity, I do not distinguish between CGM use with multiple daily injections (MDI) or insulin pumps. The benefits of CGM have been established for both, with comparatively greater benefits for MDI (e.g., Šoupal et al. 2016; Rodbard 2017). It must be noted, however, that unique advantages of using CGM in combination with a pump are (1) the function of insulin suspension when the CGM detects hypoglycemia, and (2) the potential for a hybrid closed-loop system, recently approved by the U.S. FDA, which can also administer glucagon (to raise blood glucose concentrations) when the CGM reports hypoglycemia, which further decrease chances of hypoglycemia (Rodbard 2017).

11 The normal blood glucose range is between 4–7.8 mmol/L and 72–140 mg/dL.

12 With very rapid changes in blood glucose concentrations, CGM may lag behind blood glucose testing, which is why in these cases it is advised also to consult the latter. While blood glucose testing may be more accurate in these situations, CGM alarms will still alert users to potentially dangerous blood glucose trends.

13 The severity of the risk of hypoglycemia for those with HU is why this condition currently qualifies one for CGM use in Dutch hospitals, as I will discuss in more detail later.

14 This is not to say that CGM use may not have some negative effects—just that, at present, the evidence is overwhelmingly in favor of the overall benefits significantly outweighing any potential burdens for the individual with T1D.

15 Compare this with the finger prick checking of blood glucose, which requires a blood glucose meter, a lancet, and a drop of blood from one's fingertip. This is clearly much more difficult to achieve inconspicuously.

16 There is a potential counterargument here: lower visibility of T1D-related actions will actually lead to greater stigmatization (through reduced public awareness), so that the effects of CGM will be counterproductive. While there may be some truth to this, it does not hold for the most important areas of stigmatization: work and personal relationships. Here, being able to function well appears crucial (more than visibility effects) to reducing stigma. Given that CGM is associated with better functioning (i.e., better glycemic control and fewer hypoglycemic episodes), it is ultimately more likely to reduce stigmatization.

17 It must be noted that some non-CGM forms of blood glucose measurement also offer this functionality, most notably FGM.

18 To the extent that CGM provides a more accurate representation of the state of affairs for people with T1D (that is, one's current and prospective blood glucose levels), this is very likely to enhance their ability to act intentionally in light of them, as well as increase their understanding of their situation (in the short and long term). Whether autonomy is undermined in these two senses without CGM use, however, I am not sure.

19 Emphasis mine.

20 It may also negative affect those who care for persons with T1D, potentially undermining their autonomy (e.g., if it keeps them preoccupied, unable to sleep, or keeps them from performing other activities).

21 I think that there is an interesting analogy here with providing fall detectors to elderly people who are afraid of falling, which helps them become more confident (Brownsell and Hawley 2004). Phenomenologically, (fear of) falling down and (fear of) one's blood glucose concentrations “falling” into a state of hypoglycemia, with resultant unconsciousness, appear to be closely related.

22 The mention of low blood sugar as an explanation for certain kinds of behavior (e.g., the binging of food) in films and television shows, often to comedic effect, has probably added to this notion entering popular imagination.

23 There may be, and surely are, people with T1D who are not concerned about their blood glucose levels or more generally about managing their illness. I add it as a note here that, should such individuals truly evince (repeated) unconcern for managing their illness, then this may constitute a legitimate reason for them not to be provided with CGM at the expense of others.

24 Emphasis in the original.

25 Even, or perhaps especially, if one refuses to do all or some of these things.

26 This is not to say that all will be equally invested in, affected by, or responsive to this information. One may want to avoid knowing as much as want to know.

27 High glucose values, for instance, have been shown to have a negative impact on mood for people with T1D (Hermanns et al. 2007).

28 Exactly how much glucose will depend on the needs of the individual person, knowledge of which is largely based on previous experience (which the CGM device may help to provide).

29 At least, there is no reason to assume a priori that the group currently able to purchase or receive CGM via hospitals is in any way superior in terms of their ability to know and act on that knowledge compared to those without access. Some differences may, of course, result from better or worse information provision/education about CGM use, which should be an area of concern.

30 Thanks to an anonymous reviewer for raising this objection.

31 Or, at least, not nearly as frequently. Some CGM systems still require occasional calibration using blood from finger pricks; even so, this means significantly less pricking (which, as one might imagine, is not pleasant and therefore happily avoided).

32 It is important that people with T1D retain the option not to use CGM, for they may have reasons (psychological, social, etc.) to prefer alternative ways of measuring glucose levels. Respecting autonomy entails that CGM use should not be forced, although its use should be recommended. CGM trial periods may be a fruitful way of assisting decision-making regarding whether or not CGM is preferred.

33 These are: A.S.R., CZ, Eno, DSW, IptiQ, ONVZ, Menzis, VGZ, Zilveren Kruis, and Zorg en Zekerheid. According to their website, ZN "supports its members by fulfilling the mission of the Dutch health insurers: to arrange health care of good quality for their insured, that is affordable and accessible at the same time and aimed at promoting the well-being of their insured." See: https://www.zn.nl/1483931648/About-ZN.

34 The criteria are specified on the application form for CGM/FGM devices. I have translated them into English; the original form (A) in Dutch can be found here: https://znformulieren.nl/337936417/Formulieren?folderid=338591748&title=Hulpmiddelen.

35 My translation.

36 For instance, the provision of CGM to children under the age of 18 is problematic. In itself, this is great, for children (and their caregivers) are likely to be greatly helped by CGM. However, this criterion opens up the potential of losing CGM access as soon as a child has grown up—after they have grown accustomed to it throughout their developmental years, having quite literally learned how to live with it, and thus forcing them to switch to a different and unfamiliar way of monitoring glucose. This seems decidedly unfair.

37 For more information, and the source of information for this section, see (in Dutch): https://www.glucosesensornet.nl/Content/149/vergoeding.aspx.

38 The results of cost-effectiveness analyses are typically expressed by the incremental cost-effectiveness ratio or ICER, which indicates "the money required to gain a quality-adjusted life year (QALY) (i.e., one year with best possible quality of life) at a population level," and which, as a concept, "is valuable for choosing among diverse interventions competing for limited resources" (Ioannidis and Garber  2011, p. 1).

39 All non-euro currency in this section was converted to euros using Oanda (2020) Currency Converter at the exchange rate on February 27, 2020. See: https://www1.oanda.com/currency/converter/.

40 Thanks to Marcel Verweij for this point.

41 For the complete ranking, see (in Dutch): https://www.volksgezondheidenzorg.info/ranglijst/ranglijst-aandoeningen-op-basis-van-ziektelast-dalys.

42 For the latest (2015) categorization of disease burdens, see (in Dutch) Netherlands National Institute for Public Health and the Environment (2020b).

43 Diabetes falls in category 1 of 3, with a disease burden from 0.1 up to and including 0.4.

44 Of course, findings from one setting cannot straightforwardly be extrapolated to another, so that the study by VanGenugten (2010) currently provides the best indication of cost-effectiveness in the Netherlands.
